# Successful infection of BALB/c mice by a swine hepatitis E virus clone constructed with reverse genetics

**DOI:** 10.1186/s12879-018-3544-4

**Published:** 2018-12-20

**Authors:** Wenhai Yu, Chenchen Yang, Xianhui Hao, Tianwu Ma, Fen Huang

**Affiliations:** 1 Institute of Medical Biology, Chinese Academy of Medical Sciences and Peking Union Medical College, 935 Jiaoling Road, Kunming, China; 2 Medical School, Kunming University of Science and Technology, 727 Jingming Road, Kunming, China

**Keywords:** Hepatitis E virus, Infectious cDNA clone, BALB/c mice, Infectivity

## Abstract

**Background:**

Hepatitis E virus (HEV) is a leading cause of hepatitis worldwide. However, its infection biology and pathogenesis remain largely elusive. Furthermore, no proven medication is available for treating hepatitis E. Robust experimental models are urgently required to advance the research of HEV infection. Because of the lacking of a sophisticated small animal model, this study aimed to establish a mouse model of HEV infection.

**Methods:**

We constructed a full-length swine HEV cDNA clone of genotype 4 (named as pGEM-HEV) by reverse genetics approach. And we inoculated with HEV RNA in BALB/c mice to establish small animal model for HEV infection and pathogenesis studies.

**Results:**

The capped RNA transcripts of pGEM-HEV prepared in vitro were replication-competent in HepG2 cells. Importantly, BALB/c mice intravenously inoculated with RNA transcripts of pGEM-HEV developed an active infection as shown by shedding viruses in feces, detectable negative strand of HEV in the liver, spleen and kidney, and causing liver inflammation.

**Conclusion:**

In this study, we successfully established of BALB/c mice-based small animal model for HEV provides an opportunity to further understand HEV pathogenesis and to develop effective antiviral medications.

## Background

Hepatitis E virus (HEV) is classified in the genus *Orthohepevirus* of the family *Hepeviridae* [1]. It is a non-enveloped, single-stranded, positive-sense RNA virus, with an approximately 7.3 kb genome. The viral genome consists of three open reading frames (ORFs) flanked by short 5′ and 3′ non-translated regions, ORF1 encodes a nonstructural protein, ORF2 encodes a capsid protein and ORF3 encodes a small multifunction protein that is essential for viral infection [2–5]. A unique feature as a hepatitis virus is that HEV has a zoonotic nature and can cross-species transmit in human, swine and deer [6–10].

HEV is considered the most common cause of hepatitis worldwide [11]. It causes both endemic and epidemic forms of hepatitis E in many developing countries. It is transmitted by the fecal-oral route and waterborne transmission is most often described. In developed countries, most documented cases of acute hepatitis E are sporadic and endemic cases attributed to food consumption [11–13]. Although the infection is generally acute and self-limiting, up to about 25~30% mortality has been reported following HEV infection during pregnancy [14, 15]. However, the biology and pathogenesis of HEV infection remain largely elusive and no proven antiviral medication is available.

Robust experiment models are the most important tools for advancing fundamental and translational research of hepatitis E infection. Fortunately, several cell culture systems for propagating HEV have been recently developed [16–18]. However, the development of animal models, in particular the use of small laboratory animals, has not been well-explored. Although swine and rabbit have been used to model HEV infection [19, 20], experimental infection in mouse model, the most commonly used laboratory species, has not been established.

We previously have attempted to establish BALB/c nude mice-based HEV model [21]. However, this strain lacks a thymus and is therefore unable to produce T-cells. The immunodeficient nature with a strict life condition and limited fertility has hampered the further application. To circumstance these bottlenecks, this study aimed to establish regular BALB/c mice-based HEV model. We first constructed an infectious cDNA clone of swine HEV with reverse genetics approach. We demonstrated its infectivity in cell culture and importantly also in BALB/c mice. Most interestingly, HEV provokes host response with production of anti-HEV antibodies and induction of liver inflammation, mimicking infection in human. Therefore, this model bears important implications for studying HEV infection and drug development.

## Methods

### Construction of a full-length cDNA clone of HEV

**Fig. 1:**
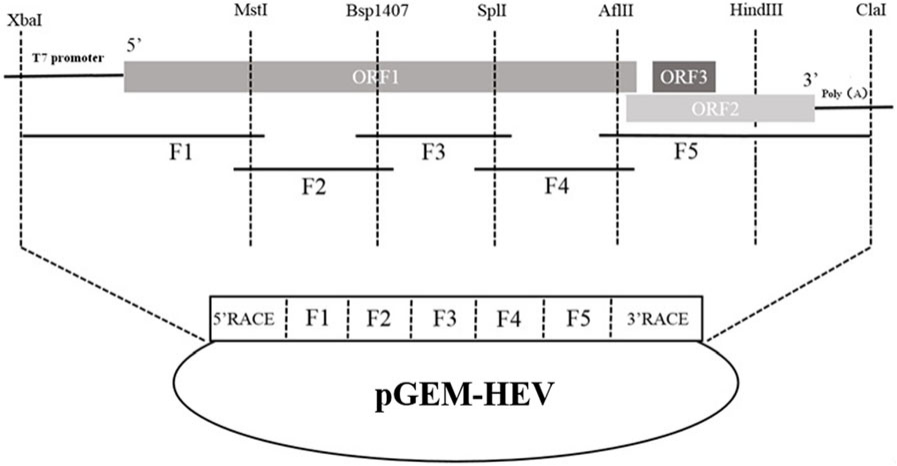
A schematic diagram of the strategy used to assemble the full-length cDNA clone of swine HEV

The full-length of swine HEV (genotype 4, KM01, GenBank No. KJ155502) was amplified with specific primers shown in Table 1 [22]. The collection of stool specimens was approved by the owner. Five overlapping fragments were amplified by PCR. The 3′ end and 5′ end of the virus were obtained using the RACE 5' or 3′ kit (Takara). The entire viral genome was ligated together at indicated restriction enzyme sites in each fragment (Fig. 1). A unique *Xba* I restriction enzyme site and a T7 RNA polymerase core promoter were introduced at the extreme 5′ terminus. Twenty-four adenosines (A) was engineered at the 3′ end of viral genome, followed by a *Cla* I restriction enzyme site for plasmid linearization (Fig. 1). PCR productions were purified and cloned into pMD-18 T vector, followed by sequencing with three clones of each fragment. The clone containing the consensus sequence was used for infectious clone assembly. One silent mutation at nucleotide (nt) 4120 (G → T) generated during the PCR amplification was retained as a genetic marker. The full-length genomic cDNA was introduced into the pGEM-7zf (+) vector (Promega) between *Xba* I and *Cla* I sites to produce a full-length HEV cDNA clone named pGEM-HEV.

**Table 1:** Primers sequence

Primer ID	Sequence (5′~ 3′)	Product	Length (bp)	Reference
S11	AGGCTCCTGGCRTYACTACTG	F1	1163 bp	2
S12	GCCYTGGCGAATGCTGTG			
A11	GGCCRGGAATGTAATCACG			
A12	GCGGCACTGGGCRTAAAACT			
HEV-A1	AAAGGAATGAAGAGGCTGGAG	F2	1520 bp	This study
HEV-A3	GAAAAGTCTGGCCGTGATTAC			
HEV-B2	TCCTCAGTAATAGTAAGGGC			
HEV-B4	AGGTCGATGGTTACGTTCCC			
HEV-C1	TGCCTGTTGGGCTGAGTTTTGATG	F3	1394 bp	This study
HEV-C3	GCCAGCCATAGCTTGGTTTGAAG			
D1R	AAGGTCTTGCTCCACGCAGATATC			
D3R	CTGGAAGAATGTTATACGAGACAC			
D2F	CTTGTGGAGGCCATGGTGGAGAA	F4	1243 bp	This study
D4F	ATGGTGGAGAAAGGCCAGGAT			
ER1	TCACGCCAAGCGGAGCCGAGT			
ER2	GAAGGGGTTGGTTGGATGAAT			
EF1	TTTCTGGGGTGACCGGGTTGATT	F5	1791 bp	This study
EF2	CTATATTCATCCAACCAACCCCT			
HEV31	CAGGGAGCGCGGAACGCAGAAAAGA			
HEV32	TCAATACTCCCGAGTTTTACCCACC			
5' Race-A1	GCAGTGARTARAGYGCAAYCCCHGTCT	5' RACE	501 bp	2
5' Race-A2	CGRGCCATYGCCTCNGCRACATC			
3' Race-S1	ACYACNACTGCTGCYACACGBTTYATGA	3' RACR	936 bp	2
3' Race-S2	CTYTGTTYAAYCTTGCTGAYACGCTKCTC			
T7P1	GCCTAGCTAGCTAGTCTAGATAATACGACTCACTATA	5′ terminal	–	This study
PAP2	CGGTCGACCGATCGATTTTTTTTTTTTTTTTTTTTTTTTTCAGGGAGCGC	3′ terminal	–	This study
HEV1	AATTATGCC(T)CAGTAC(T)CGG(A)GTTG	Positive strand of HEV RNA	348 bp	[23]
HEV2	CCCTTA(G)TCC(T)TGCTGA(C)GCATTCTC			
HEV3	GTT(A)ATGCTT(C)TGCATA(T)CATGGCT			
HEV4	AGCCGACGAAATCAATTCTGTC			
HEV6	AGCTCCTGTACCTGATGATGTTGACTC	Negative strand of HEV RNA	266 bp	[24]
HEV7	CTACAGAGCGCCAGCCTTGATTGC			
HEV8	GCTCACGTCATCTGTCGCTGCTGG			
HEV9	GGGCTGAACCAAAATCCTGACATC			
WHO-F	GGTGGTTTCTGGGGTGAC	qRT-PCR	70 bp	[25]
WHO-R	AGGGGTTGGTTGGATGAA

### In vitro transcription of capped full-length HEV genomic RNA

The full-length cDNA clones of pGEM-HEV was linearized by *Cla* I, then digested with proteinase K, and purified by phenol extraction followed by ethanol precipitation. Capped RNA transcripts from pGEM-HEV were synthesized in vitro with the T7 RiboMAX™ Express Large Scale RNA Production System (Promega, USA) according to the direction. The RNA transcripts from the cDNA clone was quantitated (1 μg /μL) and stored in − 80 °C until use.

### Cell transfection and immunofluorescence assay

The human hepatoma HepG2 cells and human lung carcinoma A549 cells were incubated at 37 °C with 5% CO_2_ in Dulbecco’s modified eagle medium (DMEM) with 10% fetal bovine serum (FBS). To evaluate the infectivity of the pGEM-HEV clone, HepG2 (60~70% confluence) cells were transfected with the capped RNA transcripts as described previously [19]. Twenty-four hours post-transfection, the cells were fixed and stained with a HEV-specific antibody (Merck Millipore, MAB8003, German, 1:1000 dilution). Briefly, cells were fixed with 4% paraformaldehyde for 15 min at 37 °C and subsequently washed three times and incubated with HEV antibody for 60 min at 37 °C. Cells were washed three times with PBS (phosphate buffered saline) and incubated with FITC (fluorescein isothiocyanate) conjugated goat anti-mouse IgG (H + L) antibody (Promega, USA, 1:1000 dilution) for 45 min at 37 °C. Nuclei were counterstained with 4′,6-diamidino-2-phenylindole (DAPI). Cells were washed three times with PBS, and viewed under a fluorescence microscopy (Nikon Ti-E, Japan).

### Inoculation of BALB/c mice with capped RNA transcripts

The protocol of animal experiments were approved by the Animal Care and Use Committee (ACUC) of Kunming University of Science and Technology. Six female, 8-week-old, SPF (specific pathogen free) BALB/c mouse purchased from SLAC laboratory animal (China), and maintained in a pathogen-free animal facility. Mice were negative to anti-HEV IgG and IgM antibodies, and HEV RNA. Six mice were randomly divided into 2 groups and each mouse was caged separately.

Group A containing Mouse 1, 2 and 3 were inoculated with RNA transcripts of the swine HEV clone pGEM-HEV (10 μg /mouse), and Group B containing mouse 4, 5 and 6 were inoculated with equal volume PBS. Feces and serum were collected every week from each mouse after inoculation. Mice were anaesthetized with pentobarbital (80 mg/kg) and euthanized by cervical dislocation at 28 days post-inoculation (dpi). Tissues, including liver, spleen, kidney and colon were collected. Animal carcasses were safely disposed by the ACUC of the Kunming University of Science and Technology (WH17037).

### Detection of HEV RNA in feces, serum and tissues by nested RT-PCR and quantitative RT-PCR

Total RNA in feces, serum and tissues were extracted by Trizol (Invitrogen), according to the manufacturer’s instructions. The isolated RNA was used to synthesize first-strand cDNA. The cDNA was added as the template to perform the reverse transcription nested PCR (RT-nPCR). Both positive strand and (or) negative strands of HEV in feces, serum and tissues were detected with strand-specific primers [23, 24] as described previously and shown in Table 1.

The viral titer of HEV in the feces, serum and tissues were quantified using SYBR green-based quantitative RT-PCR (qRT-PCR) with HEV-specific primers [25]. qRT-PCR was performed using the BIO-RAD CFX Connect Real-Time System under the following conditions: 95 °C for 30 s, followed by 39 cycles of 95 °C for 5 s and 60 °C for 31 s.

### Determination of HEV antibodies by ELISA

The HEV IgG and IgM antibodies in serum were tested by ELISA (KHB, China) according to the manufacturer’s instructions. The cutoff values for the IgG and IgM assay were determined based on 0.22 (or 0.24 for IgM) plus the mean OD450/630 values of serum from uninfected mouse (± standard deviation, S. D.).

### Profile liver biochemistry in serum

The activities of alanine aminotransferase (ALT), aspartate aminotransferase (AST) and alkaline phosphatase (ALP) in serum were measured with an automated biochemistry analyzer (Olympus 2700, Japan).

### Detection of HEV ORF2 protein in tissues by Western blotting

The livers, spleens, kidneys and colons were collected and HEV capsid protein were detected in these tissues by Western blotting. Briefly, the tissues were broken in liquid nitrogen by grinding, and were lysed in RIPA buffer. Equal volumes of tissue lysates from each condition were resolved by 10% sodium dodecyl sulfate-polyacrylamide gel electrophoresis (SDS-PAGE). Analysis of protein was visualized by direct Western blotting using antibodies directed against the indicated antigens.

### Infection of A549 cells with viruses recovered from mouse inoculated with capped RNA transcripts of swine HEV clones

The mouse feces were suspended into 0.1% DEPC (diethyl pyrocarbonate)-PBS. The supernatant was collected by centrifuge and microorganisms were removed by filter. A549 cells were infected with the viruses recovered from mice experimentally infected with feces supernatant. Cells were harvested at 72 h post-infection, and the HEV ORF2 protein was be detected by Western blotting.

### Histopathologic examination

The liver tissues were fixed in 10% neutral buffered formalin, sectioned at a thickness of 3 μm, and stained with hematoxylin and eosin. All sections were examined using a Nikon Ti-E microscope (Japan).

### Statistical analysis

Prism software (GraphPad Software) was used for statistical analysis. Data were presented as mean ± S. D..

## Results

### A full-length cDNA clone of swine HEV is infectious

**Fig. 2:**
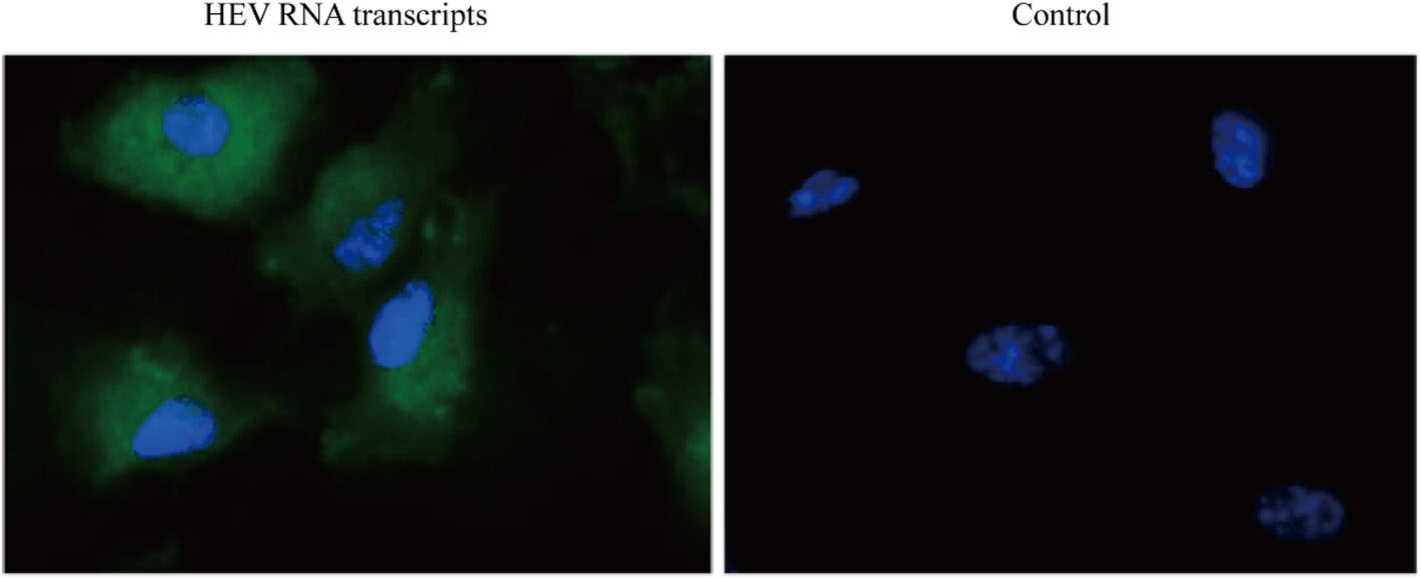
Infectivity of pGEM-HEV in HepG2 cells identified by IFA. Immunofluorescence staining with anti-HEV ORF2 antibody in HepG2 cells transfected with capped full-length RNA transcripts pGEM-HEV (HEV RNA transcripts) and non-transfected control cells (Control). Nuclei were counterstained with DAPI

To assess the infectivity of the pGEM-HEV cDNA clone, capped RNA transcripts were transfected into HepG2 cells. Twenty-four hours post-transfection, HEV antigens were detected by immunofluorescence assay (IFA) with an anti-HEV ORF2 monoclonal antibody. The intense fluorescent signals of HEV antigens indicating that the transfected viral RNA was replication-competent in HepG2 cells (Fig. 2). The fluorescent signal was detected in HepG2 cells transfected with the capped RNA transcripts, while no signal in non-tranfected control cells.

### Capped RNA transcripts of pGEM-HEV was infectious in BALB/c mice

**Fig. 3:**
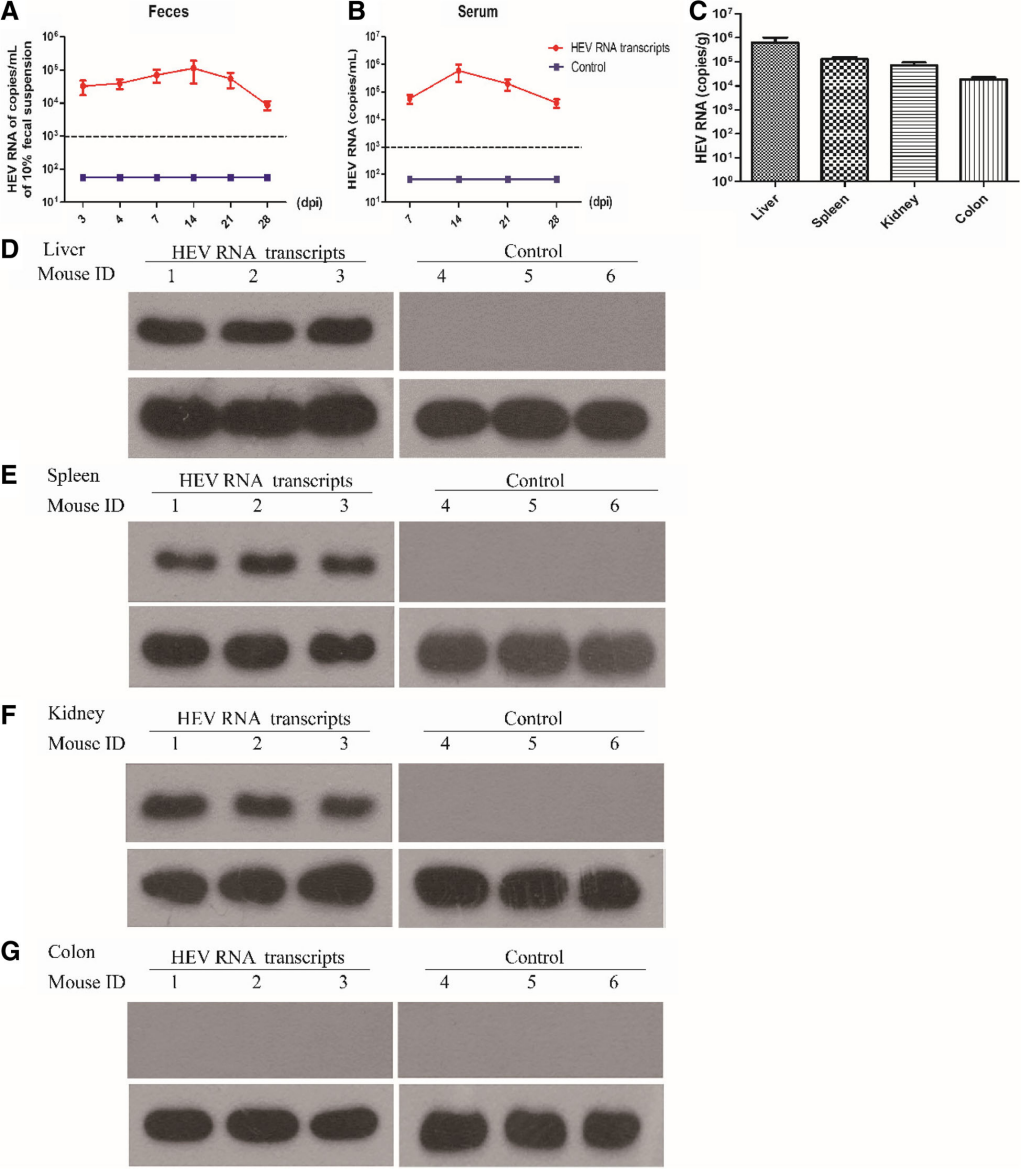
HEV viral titer and HEV capsid protein detection. The viral titer of HEV in feces (**a**), serum (**b**) and tissues (**c**) were detected by qRT-PCR. HEV capsid protein was detected in different tissues of BALB/c mouse, liver (**d**), spleen (**e**), kidney (**f**) and colon (**g**) by Western blotting

To further investigate whether this clone can be used to establish infection in mice, we explored the infection in BALB/c mouse model. Mice in groups A and B were intravenously injected with full-length capped RNA transcripts from pGEM-HEV (HEV RNA transcripts) and PBS (Control), respectively. HEV RNA was first detected in feces at 3 dpi in group A inoculated with RNA transcripts, and it was all detected in feces from three mice feces after 7 dpi, and lasted to the end of the experiment (Table 2). The viral titer of HEV in the feces of mice inoculated with HEV RNA transcripts was detected from 3 to 28 dpi (Fig. 3a). In the serum, HEV RNA positive-strands were all detected in mice inoculated with RNA transcripts from 7 to 28 dpi (Fig. 3b). The HEV negative-strand RNA was detected in one mouse at 7 dpi and two mice at 14 dpi in group A inoculated with RNA transcript, and all detected at 21 dpi. However, mice injected with PBS were negative to both positive and negative strands during the whole experiment.

**Table 2:** Detection of HEV RNA by RT-nPCR in feces, serum and tissues of mouse inoculated with capped full-length RNA transcripts of pGEM-HEV or PBS

Sample		Strand	HEV RNA transcripts	Control
Feces	0 dpi	Positive	–	–
	3 dpi	Positive	+(1/3)	+(0/3)
	4 dpi	Positive	+(2/3)	+(0/3)
	7 dpi	Positive	+(3/3)	+(0/3)
	14 dpi	Positive	+(3/3)	+(0/3)
	21 dpi	Positive	+(3/3)	+(0/3)
	28 dpi	Positive	+(3/3)	+(0/3)
Serum	0 dpi	Positive	+(0/3)	+(0/3)
		Negative	+(0/3)	+(0/3)
	7 dpi	Positive	+(3/3)	+(0/3)
		Negative	+(1/3)	+(0/3)
	14 dpi	Positive	+(3/3)	+(0/3)
		Negative	+(2/3)	+(0/3)
	21 dpi	Positive	+(3/3)	+(0/3)
		Negative	+(3/3)	+(0/3)
	28 dpi	Positive	+(3/3)	+(0/3)
		Negative	+(2/3)	+(0/3)
Tissues	Liver	Positive	+(3/3)	+(0/3)
		Negative	+(3/3)	+(0/3)
	Spleen	Positive	+(3/3)	+(0/3)
		Negative	+(3/3)	+(0/3)
	Kidney	Positive	+(2/3)	+(0/3)
		Negative	+(2/3)	+(0/3)
	Colon	Positive	+(0/3)	+(0/3)
		Negative	+(1/3)	+(0/3)

Replication of HEV in multiple tissues, including liver, spleen, kidney and colon, has been reported in both pigs and nude mice when inoculated with HEV [21, 26]. In order to further analyze the replication of HEV in different tissues, HEV genome RNA and capsid protein (ORF2) were analyzed by RT-nPCR, qRT-PCR and Western blotting, respectively. In the present study, HEV RNA was detected with a high viral titer in the liver (6.2 × 10^5^ ± 5.8 × 10^4^ copies/g), spleen (1.3 × 10^4^ ± 3.7 × 10^4^ copies/g), kidney (7.1 × 10^4^ ± 3.1 × 10^4^ copies/g) and colon (1.9 × 10^4^ ± 5.8 × 10^3^ copies/g) of mice inoculated with HEV RNA transcripts at 28 dpi (Fig. 3c). HEV RNA (both positive and negative strands) were detected in the liver and spleen in mice inoculated with HEV RNA transcripts at 28 dpi (Table 2). Although colon has been reported as a replicate site of HEV [21], only one mouse inoculated with HEV RNA transcripts at 28 dpi was found to be positive (Table 2). Meanwhile, the capsid protein of HEV (ORF2) were detected in the liver, spleen and kidney of mice inoculated with HEV RNA transcripts (Fig. 3d-g) by Western blotting. Shedding viruses in the feces, detected HEV RNA antigens in the serum and tissues indicated that HEV RNA transcripts was infectious in BALB/c mice.

### BALB/c mice produce infectious HEV viruses upon inoculation of capped RNA transcripts of the swine HEV clone

**Fig. 4:**
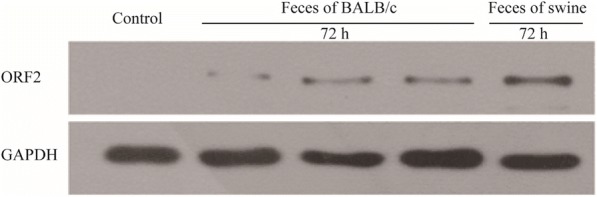
BALB/c mice produce infectious HEV viruses upon inoculation of capped RNA transcripts of the swine HEV clone. A549 cells were infected with the feces suspension of BALB/c mouse inoculated with HEV RNA transcripts. Cells were harvested at 72 h post-inoculation to detect ORF2 protein by Western blotting. The feces suspension of swine HEV served as positive control. GAPDH served as a loading control

To confirm whether BALB/c mice can produce infectious HEV viruses, we attempted to infect A549 cells with the recovered viruses from mice inoculated with capped RNA transcripts of the swine HEV clone. A549 cells were harvested 72 h after infection. HEV capsid protein was detected by Western blotting (Fig. 4) in cells infected with the feces of BALB/c mice inoculated with HEV RNA transcripts. This result confirmed that the inoculated BALB/c mice can produce infectious HEV, which can replicate in A549 cells.

### Capped RNA transcripts of pGEM-HEV provokes humoral response in BALB/c mice

**Fig. 5:**
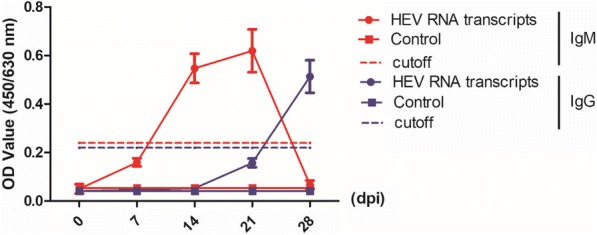
Anti-HEV IgM and IgG antibodies in BALB/c mouse were tested by ELISA. Anti-HEV IgM and IgG antibodies in BALB/c mouse inoculated with HEV RNA transcripts or PBS were tested by ELISA

In response to HEV infection, human body develops antibodies to HEV (IgM and IgG). To investigate whether BALB/c mice provoke similar response to RNA transcripts of HEV infection, we measured serum anti-HEV IgM and IgG. An increased anti-HEV IgM and IgG levels were observed in all the mice inoculated with HEV RNA transcripts compared with the control group (Fig. 5). In the RNA transcripts inoculated group, the increased IgM level reached the pink at 21 dpi. IgG began to increase at 21 dpi, and continued to increase until the end of the experiment. These results demonstrated that infection of the swine HEV infectious clone stimulated humoral response in BALB/c mice.

### Infection of capped HEV RNA transcripts induces liver inflammation in BALB/c mice

**Fig. 6:**
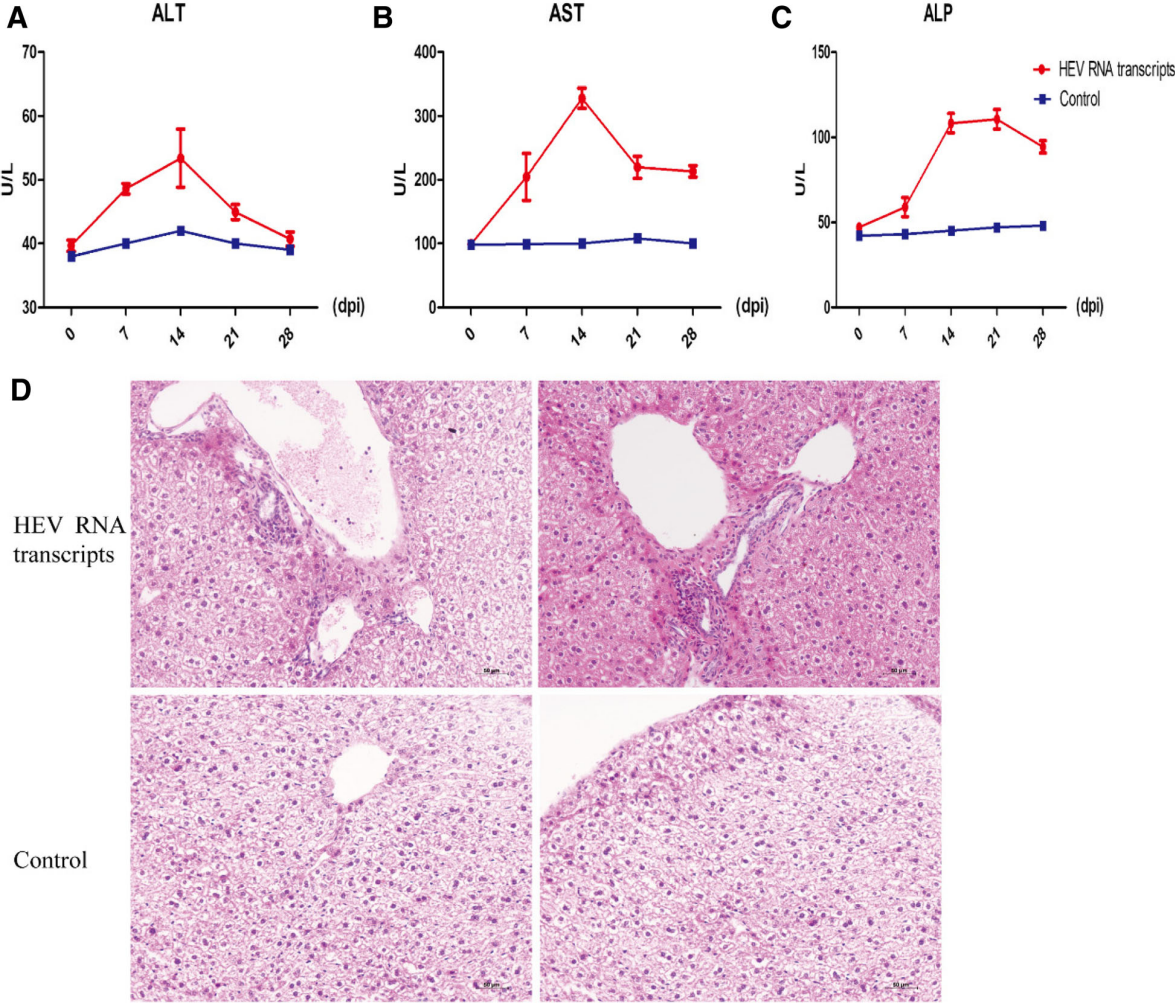
The activities of liver-specific enzymes and the pathological changes in BALB/c mouse inoculated with HEV RNA transcripts of pGEM-HEV. The level/activity of ALT (**a**), AST (**b**) and ALP (**c**) were determined in BALB/c mouse inoculated with HEV RNA transcripts or PBS. Histopathological analyses of the liver of BALB/c mice inoculated with HEV RNA transcripts of pGEM-HEV (HEV RNA transcripts) or PBS (Control) stained with H&E (**d**)

Hepatitis is swelling and inflammation of the liver, and most commonly caused by hepatitis virus infections. To determine whether infection of capped HEV RNA transcripts can also cause liver injury/inflammation in BALB/c mice, we first tested the widely used liver enzymes for detecting liver damage. The levels/activities were characterized using an automated biochemistry analyzer. To our surprise, the elevated ALT, AST and ALP levels/activities in BALB/c inoculated with HEV RNA transcripts was very similar to the clinical feature of HEV infected patients (Fig. 6a, b and c).

Furthermore, histopathologic examination showed swelling of liver cells and liver capsules were filled with inflammatory exudates and liver hemorrhage in mice inoculated with RNA transcripts of HEV (Fig. 6d). Increased infiltrating lymphocytes and macrophages were observed in these mice. In contrast, no damage was observed in any of the control tissues. These results indicate that infection of swine HEV RNA transcripts triggered liver inflammation in BALB/c mice.

## Discussions

Lessons from the research of hepatitis B and C viruses have taught us the importance of developing robust experimental models for understanding their infection biology, pathogenesis, as well as antiviral drug development. However, the restricted tropism of host and cell type for hepatitis virus infection has always challenged the development of experimental models. Despite the recent establishment of several cell culture systems of HEV [27], animal models are urgently need for studying HEV pathogenicity and antiviral drug development.

Non-human primates have been successfully employed for HEV research in early days [28, 29], but non-human primates are not available for research anymore, because of the ethics. Although swine is the mainly reservoir of HEV, it is not suitable as an experimental model for HEV because of its large body size. In contrast, mice are the most commonly used small animal for research purpose. This study, we reported the successful establishment of HEV infection in BALB/c mice with an infectious swine HEV clone generated by reverse genetics approach.

In fact, we have previously attempted to establish HEV mouse model using BALB/c nude mice [21]. Because nude mice lack the thymus and are therefore unable to produce T-cells. We thought that the immunodeficient nature of these mice could be an advantage of permitting HEV infection. Indeed, we have succeeded the infection in BALB/c nude mice with genotype 4 swine HEV strain [21]. However, the immunodeficient property in turn hampered the study of HEV pathogenesis. Because hepatitis is an inflammatory process mainly mediated by host immune response. Furthermore, these mice have a strict life condition and limited fertility, which also hampers the further application. To circumstance these limitations, we now have succeeded with infecting the regular BALB/c mice, which have a competent immune system. In the present study, the RNA transcripts of infectious cDNA clone of swine HEV was successfully infected BALB/c mice. HEV RNA was detected at 3 dpi in feces, which is similar to our genotype 4 swine HEV strain inoculated into BALB/c nude mice [21], but earlier than that inoculated pigs with genotype 4 swine HEV RNA transcripts (7 dpi) [20] and *Sprague-Dawley* rat (SD rat) inoculated with genotype 4 swine HEV RNA transcripts [30]. HEV produces an intermediate negative-strand RNA when it replicates. The negative-strand of HEV was detectable at 7 dpi in serum of all mice inoculated with RNA transcripts. Furthermore, the negative-strand was found in the HEV replicated sites, including liver (3/3), spleen (3/3), kidney (2/3) and colon (1/3). Moreover, HEV RNA was detected in the feces, serum and tissues (liver, spleen, kidney and colon). Shedding HEV in feces, and detection of negative-strand RNA in liver, spleen and kidney firmly demonstrated the infectivity of the genotype 4 swine HEV pGEM-HEV cDNA clone is infectious in BALB/c mice, although previous study reported that C57BL/6 mice is not permissive for HEV infection [31]. More interestingly, we observed humoral response and liver injury in infected BALB/c mice, indicating that this model is promising for studying HEV pathogenesis.

Although HEV infects BALB/c mice has been successfully established, key parameters should be determined in the future, such as identification of infected cell populations, observation of inflammatory manifestation, and then explicit comparisons with HEV infected patients to assess potential for clinical relevance. The establishment of HEV infection based on BALB/c mice is benefit to screen anti-HEV drugs and develop vaccines in vivo.

## Conclusion

In this study, we successfully established BALB/c mice-based animal model with an infectious cDNA clone of genotype 4 swine HEV constructed by reverse genetics approach. This swine HEV clone strain is capable of modeling HEV infection, and studying host response and pathogenesis. Furthermore, this model also bears importantly implications for future anti-HEV drug development.

## Abbreviations

A549 cells: Human lung carcinoma cell line
ACUC: Animal Care and Use Committee
ALP: Alkaline phosphatase
ALT: Alanine aminotransferase
AST: Aspartate aminotransferase
ATCC: American Type Culture Collection
DAPI: 4′,6-diamidino-2-phenylindole
DEPC: Diethyl pyrocarbonate
DMEM: Dulbecco’s modified eagle medium
dpi: days post-inoculation
ELISA: Enzyme-linked immunosorbent assay
FBS: Fetal bovine serum
FITC: Fluorescein isothiocyanate
HepG2 cells: Human hepatocellular carcinoma cell line
HEV: Hepatitis E virus
IFA: Immunofluorescence assay
IgG: Immunoglobulin G
IgM: Immunoglobulin M
ORFs: Open reading frames
PBS: Phosphate buffered saline
qRT-PCR: quantitative real time PCR
RACE: Rapid Amplification of cDNA Ends
RT-nPCR: Reverse transcription nested PCR
S. D.: Standard deviation
SD rat: *Sprague-Dawley* rat
SDS-PAGE: Sodium dodecyl sulfate-polyacrylamide gel electrophoresis
SPF: Specific pathogen free

## References

[1] Smith DB, Simmonds P, Jameel S, Emerson SU, Harrison TJ, Meng XJ, Okamoto H, Van der Poel WH, Purdy MA (2014). Consensus proposals for classification of the family Hepeviridae. J Gen Virol.

[2] Billam P, Sun ZF, Meng XJ (2007). Analysis of the complete genomic sequence of an apparently avirulent strain of avian hepatitis E virus (avian HEV) identified major genetic differences compared with the prototype pathogenic strain of avian HEV. J Gen Virol.

[3] Panda SK, Nanda SK, Zafrullah M, Ansari IH, Ozdener MH, Jameel S (1995). An Indian strain of hepatitis E virus (HEV): cloning, sequence, and expression of structural region and antibody responses in sera from individuals from an area of high-level HEV endemicity. J Clin Microbiol.

[4] Huang YW, Opriessnig T, Halbur PG, Meng XJ (2007). Initiation at the third in-frame AUG codon of open reading frame 3 of the hepatitis E virus is essential for viral infectivity in vivo. J Virol.

[5] Tam AW, Smith MM, Guerra ME, Huang CC, Bradley DW, Fry KE, Reyes GR (1991). Hepatitis E virus (HEV): molecular cloning and sequencing of the full-length viral genome. Virology.

[6] Meng XJ, Purcell RH, Halbur PG, Lehman JR, Webb DM, Tsareva TS, Haynes JS, Thacker BJ, Emerson SU (1997). A novel virus in swine is closely related to the human hepatitis E virus. Proc Natl Acad Sci U S A.

[7] Hsieh SY, Meng XJ, Wu YH, Liu ST, Tam AW, Lin DY, Liaw YF (1999). Identity of a novel swine hepatitis E virus in Taiwan forming a monophyletic group with Taiwan isolates of human hepatitis E virus. J Clin Microbiol.

[8] Feagins AR, Opriessnig T, Huang YW, Halbur PG, Meng XJ (2008). Cross-species infection of specific-pathogen-free pigs by a genotype 4 strain of human hepatitis E virus. J Med Virol.

[9] Feagins AR, Opriessnig T, Guenette DK, Halbur PG, Meng XJ (2007). Detection and characterization of infectious hepatitis E virus from commercial pig livers sold in local grocery stores in the USA. J Gen Virol.

[10] Tei S, Kitajima N, Takahashi K, Mishiro S (2003). Zoonotic transmission of hepatitis E virus from deer to human beings. Lancet.

[11] Rein DB, Stevens GA, Theaker J, Wittenborn JS, Wiersma ST (2012). The global burden of hepatitis E virus genotypes 1 and 2 in 2005. Hepatology.

[12] Dalton HR, Bendall R, Ijaz S, Banks M (2008). Hepatitis E: an emerging infection in developed countries. Lancet Infect Dis.

[13] Aggarwal R, Krawczynski K (2000). Hepatitis E: an overview and recent advances in clinical and laboratory research. J Gastroenterol Hepatol.

[14] Khuroo MS, Kamili S (2003). Aetiology, clinical course and outcome of sporadic acute viral hepatitis in pregnancy. J Viral Hepat.

[15] Kumar A, Beniwal M, Kar P, Sharma JB, Murthy NS (2004). Hepatitis E in pregnancy. Int J Gynaecol Obstet.

[16] Shukla P, Nguyen HT, Torian U, Engle RE, Faulk K, Dalton HR, Bendall RP, Keane FE, Purcell RH, Emerson SU (2011). Cross-species infections of cultured cells by hepatitis E virus and discovery of an infectious virus-host recombinant. Proc Natl Acad Sci U S A.

[17] Shukla P, Nguyen HT, Faulk K, Mather K, Torian U, Engle RE, Emerson SU (2012). Adaptation of a genotype 3 hepatitis E virus to efficient growth in cell culture depends on an inserted human gene segment acquired by recombination. J Virol.

[18] Tanaka T, Takahashi M, Takahashi H, Ichiyama K, Hoshino Y, Nagashima S, Mizuo H, Okamoto H (2009). Development and characterization of a genotype 4 hepatitis E virus cell culture system using a HE-JF5/15F strain recovered from a fulminant hepatitis patient. J Clin Microbiol.

[19] Huang YW, Haqshenas G, Kasorndorkbua C, Halbur PG, Emerson SU, Meng XJ (2005). Capped RNA transcripts of full-length cDNA clones of swine hepatitis E virus are replication competent when transfected into Huh7 cells and infectious when intrahepatically inoculated into pigs. J Virol.

[20] Cordoba L, Feagins AR, Opriessnig T, Cossaboom CM, Dryman BA, Huang YW, Meng XJ (2012). Rescue of a genotype 4 human hepatitis E virus from cloned cDNA and characterization of intergenotypic chimeric viruses in cultured human liver cells and in pigs. J Gen Virol.

[21] Huang F, Zhang W, Gong G, Yuan C, Yan Y, Yang S, Cui L, Zhu J, Yang Z, Hua X (2009). Experimental infection of Balb/c nude mice with hepatitis E virus. BMC Infect Dis.

[22] Fu H, Wang L, Zhu Y, Geng J, Li L, Wang X, Bu Q, Zhuang H (2011). Analysing complete genome sequence of swine hepatitis E virus (HEV), strain CHN-XJSW13 isolated from Xinjiang, China: putative host range, and disease severity determinants in HEV. Infect Genet Evol.

[23] Huang FF, Haqshenas G, Guenette DK, Halbur PG, Schommer SK, Pierson FW, Toth TE, Meng XJ (2002). Detection by reverse transcription-PCR and genetic characterization of field isolates of swine hepatitis E virus from pigs in different geographic regions of the United States. J Clin Microbiol.

[24] Nanda SK, Panda SK, Durgapal H, Jameel S (1994). Detection of the negative strand of hepatitis E virus RNA in the livers of experimentally infected rhesus monkeys: evidence for viral replication. J Med Virol.

[25] Baylis SA, Blumel J, Mizusawa S, Matsubayashi K, Sakata H, Okada Y, Nubling CM, Hanschmann KM (2013). World Health Organization international standard to harmonize assays for detection of hepatitis E virus RNA. Emerg Infect Dis.

[26] Huang F, Hua X, Yang S, Yuan C, Zhang W (2009). Effective inhibition of hepatitis E virus replication in A549 cells and piglets by RNA interference (RNAi) targeting RNA-dependent RNA polymerase. Antivir Res.

[27] Okamoto H (2011). Hepatitis E virus cell culture models. Virus Res.

[28] Aggarwal R, Kamili S, Spelbring J, Krawczynski K (2001). Experimental studies on subclinical hepatitis E virus infection in cynomolgus macaques. J Infect Dis.

[29] Panda SK, Ansari IH, Durgapal H, Agrawal S, Jameel S (2000). The in vitro-synthesized RNA from a cDNA clone of hepatitis E virus is infectious. J Virol.

[30] Zhu Y, Yu X, Zhang Y, Ni Y, Si F, Yu R, Dong S, Huang Y, Li Z (2013). Infectivity of a genotype 4 hepatitis E virus cDNA clone by intrahepatic inoculation of laboratory rats. Vet Microbiol.

[31] Li TC, Suzaki Y, Ami Y, Tsunemitsu H, Miyamura T, Takeda N (2008). Mice are not susceptible to hepatitis E virus infection. J Vet Med Sci.

